# Compressive properties analysis of mono-sized fragmented coal and rock

**DOI:** 10.1038/s41598-025-99964-z

**Published:** 2025-05-23

**Authors:** Dingyi Hao, ShiKun Xu, Shihao Tu, Hongbin Zhao, Long Tang

**Affiliations:** 1 School of Safety Science and Engineering, Anhui University of Science & Technology, Huainan, 232001 Anhui China; 2 State Key Laboratory for Safe Mining of Deep Coal Resources and Environment Protection, Huainan, 23200 Anhui China; 3 Key Laboratory of Deep Coal Resource Mining Ministry of Education, School of Mines, China University of Mining and Technology, Xuzhou, 221116 Jiangsu China

**Keywords:** Fragmented coal and rock, Mono-sized, Compressive properties, Porosity evolution, Acoustic emission characteristics, Fossil fuels, Civil engineering

## Abstract

The compressive properties of fragmented coal and rock aggregates within goaf critically influences surface subsidence dynamics and reservoir development strategies. This study conducted constrained compression testing on mono-sized fragmented coal and rock particulate materials using a custom-designed compaction apparatus, evaluating parameters including compressive resistance, pre-/post-compaction mass variations, porosity evolution, and acoustic emission (AE) signatures. Experimental observations demonstrated progressive yet decelerating axial strain development under increasing stress, accompanied by diminishing porosity reduction rates. AE activity exhibited proportional escalation in both event frequency and energy release intensity during particle consolidation. Particle dimension inversely correlated with compaction strength, while fragmented coal generated higher AE responses compared to rock counterparts. Three distinct compression phases were identified: void compaction, pore compaction, and particle recombination. These findings establish mechanistic insights for optimizing goaf flow field modeling and backfill mining techniques through enhanced understanding of energy dissipation patterns in particulate media.

## Introduction

Mining-induced overburden collapse forms distinct structural zones, among which the fragmented coal-rock mixtures in the caving zone develop heterogeneous geometries and size distributions^1^. These aggregates regain load-bearing capacity under compaction, stabilizing overlying strata and creating stress-recovery zones with water storage potential in goafs^2^. Understanding their compressive properties is critical for subsidence mitigation and reservoir development.

Recent investigations employing diverse methodologies-primarily discrete element modeling (DEM)-have advanced compressive properties analysis. Studies using three-dimensional discrete element code (3DEC)^3^ and particle flow code three-dimensional (PFC3D)^4,5^ simulated particle interactions and stress transfer, revealing confining pressure’s role in enhancing gangue-bearing capacity. Zhang et al. quantified pore-fracture evolution under varying coal-rock ratios^6–8^, while Huang et al. modeled joint-strength impacts on stress–strain behavior^9^. Random crushed stone model based on Matrix Laboratory-Particle Flow Code (MATLAB-PFC)^10^ and elastic theory-based frameworks^5^ further elucidated size-dependent compressive dynamics.

Laboratory studies complement simulations: Ma et al. identified particle size and lithology as key determinants of stress–strain relationships^11–13^, corroborated by Wen et al. through acoustic emission (AE)-monitored compression stages^14^. Fractal analysis quantified fragmentation patterns^15,16^, while cubic stress functions described porosity-compaction correlations^17,18^. Multi-factor isotropic tests^19^ and thermal–hydraulic models^20,21^ expanded parametric understanding, with triphasic compaction stages^22^ aligning with field observations^23,24^. Hybrid approaches integrating simulations and experiments enabled 3D modeling of particle re-crushing and porosity inversion^25–29^.

However, existing studies lack synchronized analysis of stress, porosity, mass variation, and AE characteristics during confined compaction. Addressing this gap, our research systematically examines mono-lithology, single-size coal and rock aggregates under controlled loading, evaluating bulking coefficients, compaction strength, pre-/post-compaction mass variations, porosity evolution, and AE signatures. This integrated approach enhances mechanistic insights into goaf stability and reservoir engineering.

## Sample preparation and determination of bulking coefficient

### Preparation of coal and rock samples

A large volume of coal was sampled from the 30,105 working face of the 3–1 coal seam in Nanliang Mine, and the core (including medium sandstone, fine sandstone, and siltstone) with a diameter of 70 mm was drilled from the top and bottom of the working face, wrapped with plastic film, and finally transported to the coal sample processing location.

**Fig. 1:**
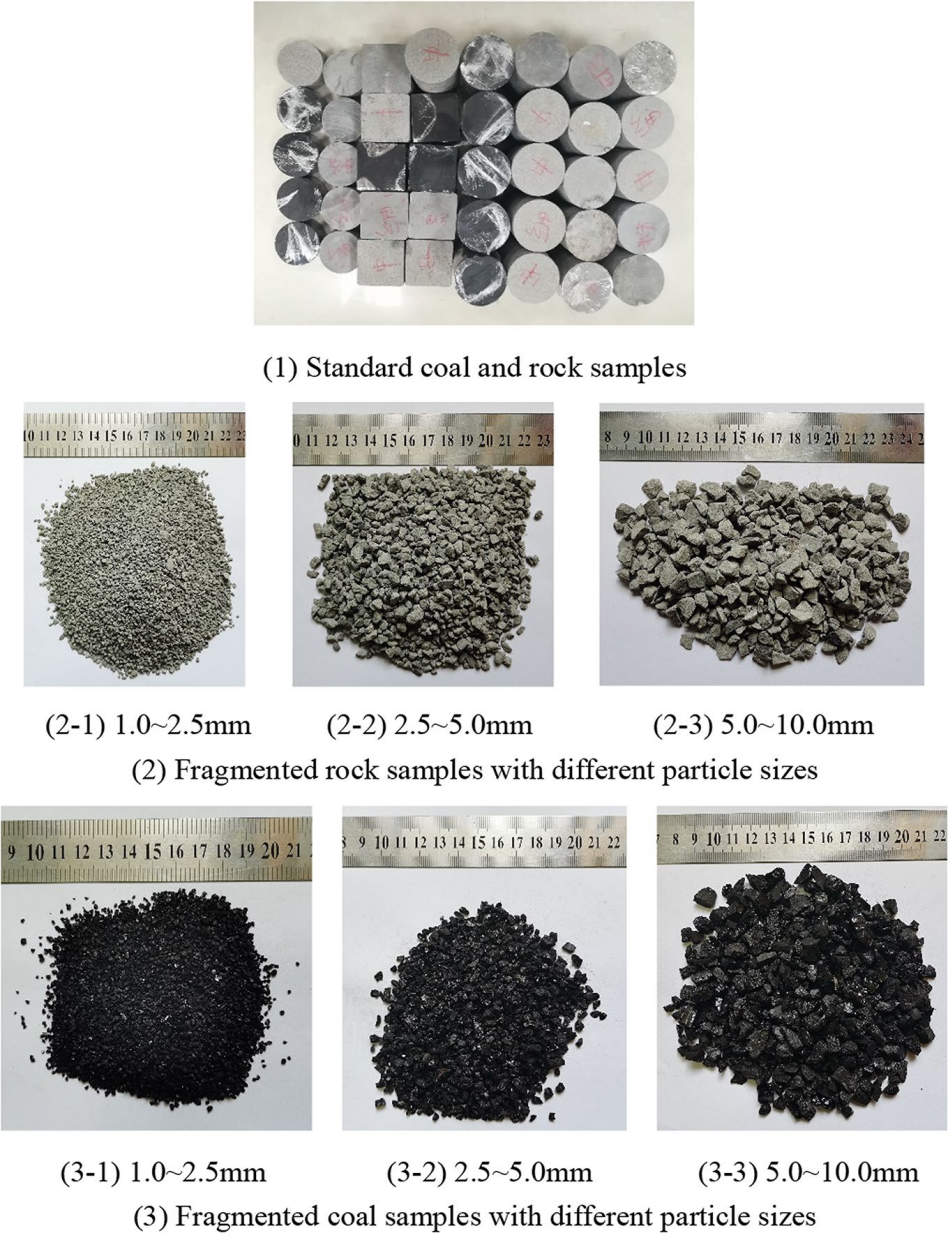
Standard and fragmented coal and rock samples with different particle sizes.

Standard coal and rock samples were processed (as shown in Fig. 1(1)). First, a uniaxial compressive test, a Brazilian splitting tensile test, and an angular compression shear test were performed to determine the corresponding mechanical parameters of the above samples. The cylindrical test apparatus with an internal diameter (D) of 50 mm imposed a maximum particle size (d) of 10 mm for fragmented coal-rock specimens, adhering to the geometric constraint D/d > 5 as per rock mechanics standards. Fractured coal and rock particles were mechanically sieved into three granulometric fractions: 1.0–2.5, 2.5–5.0, and 5.0–10.0 mm using high-frequency vibratory separation. Representative samples of each size class are visually documented in Fig. 1.

**Fig. 2:**
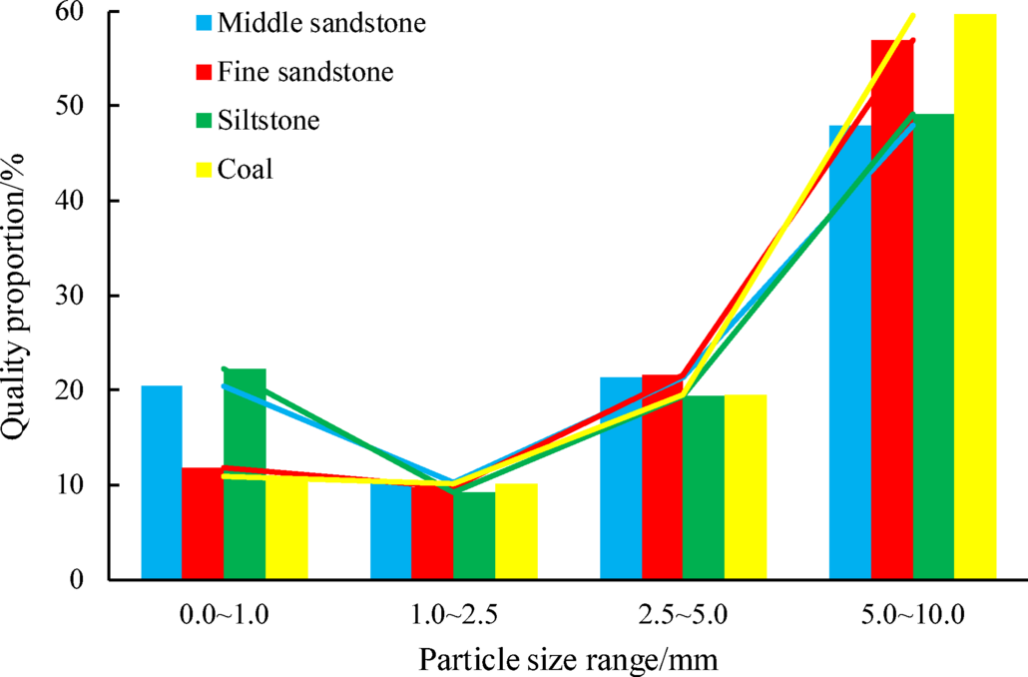
Mass proportion of each particle size after crushing of different coal and rock masses.

Mechanical crushing of middle sandstone, fine sandstone, siltstone, and coal yielded distinct granulometric distributions (Tables 1 and Fig. 2). Analysis revealed a bimodal particle size trend: the 5.0–10.0 mm fraction dominated across lithologies, while smaller fractions (0.0–1.0 mm) exhibited unexpectedly higher mass percentages than the 1.0–2.5 mm class. The sub-1.0 mm particles, predominantly pulverized material unsuitable for compaction or permeability testing, were excluded from subsequent analysis.

**Table 1:** Quality and proportion of fragmented rock and coal with different particle sizes.

Lithology	0.0–1.0 mm	1.0–2.5 mm	2.5–5.0 mm	5.0–10.0 mm	Total mass (kg)
Middle sandstone	2.10	1.06	2.18	4.92	10.25
	20.44%	10.32%	21.29%	47.95%	100%
Fine sandstone	2.07	1.71	3.79	9.98	17.54
	11.79%	9.72%	21.61%	56.88%	100%
Siltstone	4.45	1.83	3.88	9.79	19.95
	22.28%	9.22%	19.43%	49.07%	100%
Coal	264.8	245.6	473.8	1452.4	2436.6
	10.86%	10.08%	19.45%	59.61%	100%

### Bulking coefficient of fragmented coal and rock mass

Given the axial pressure-dependent nature of particulate coal-rock bulking coefficients^30^, pre-compaction measurement of this parameter across particle size fractions is essential. The coefficient is calculated as:

1 $$c_{b} = \frac{{V_{0}^{\prime } }}{{V_{0} }}$$

Here, *V*_0_ and $V_{0}^{\prime }$ are the volume of the rock before and after crushing, respectively, mm^3^.

Since the quality of the rock before and after crushing is fixed, the bulking coefficient of the fragmented rock can be obtained after placing the fragmented rock in a container with the same diameter as the intact elastic standard rock sample:

2 $$c_{b} = \frac{{h_{y} m_{1} }}{{m_{2} h_{0} }}$$

Here, *c*_*b*_ is the bulking coefficient, *m*_1_ and *m*_2_ are the quality of the intact elastic standard rock sample (φ50 × 100 mm) and the quality of the fragmented rock, respectively; *h*_0_ and *h*_y_ are the heights corresponding to the intact elastic standard rock sample *m*_1_ and the fragmented rock (bottom diameter of 50 mm) *m*_2_ before confined compaction, respectively.

A complete elastic standard coal rock sample (φ50 × 100 mm) was considered as the research subject. Fragmented rock sample preparation involved normalizing particulate materials to a 250 g equivalent height (constant base area) through mass-height calibration to determine initial bulking coefficients (fragmented rock sample is 150 g). Uniformly blended mono-sized fragmented rock underwent eight replicate measurements, with mean values tabulated (Table 2). The inverse correlation between particle size range and bulking coefficient aligns with Miao et al.'s particle-size dependency observations^30^.

**Table 2:** Bulking coefficient of mono-sized fragmented coal and rock.

Particle size range (mm)	Siltstone	Fine sandstone	Middle sandstone	Coal
1.0–2.5	2.01	2.02	1.89	2.04
2.5–5.0	2.04	2.00	1.86	2.02
5.0–10.0	2.00	1.94	1.80	1.97

## Compressive properties of fragmented rock with mono-sized

Four key parameters were analyzed in the fragmented coal and rock mass densification process: compressive strength, pre-/post-compaction mass variation across particle size fractions, porosity evolution, and acoustic emission signatures.

### Compaction strength

**Fig. 3:**
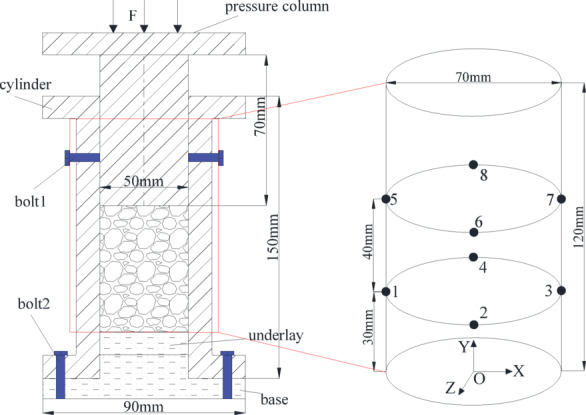
Lateral pressure limiting compaction device and acoustic emission positioning point for CT scanning of in-situ fragmented rock pores.

A specialized lateral pressure limiting compaction device (Fig. 3) was developed for particulate coal-rock analysis, modifying Yu et al.'s design to integrate real-time CT-compatible functionality^31^. Constructed from high-temperature-resistant (260 °C) nylon with superior wear resistance and mechanical stability, this cost-effective apparatus prevents particle dispersion during compression through radial constraint. Bolt 1 maintains structural integrity post-compaction, while the material’s radiolucency ensures X-ray transparency for real-time pore imaging throughout densification processes.

The cylinder wall thickness of the side-limited compaction device was 10 mm, and the stiffness was high; therefore, the radial deformation of the cylinder in the loading process could be ignored. When axial loading was conducted, the fragmented coal and rock mass can be considered as a whole, and the axial stress–strain are the stress and strain of the entire structure. The axial stress can be expressed as follows:

3 $$\sigma_{y} { = }\frac{{4F_{b} }}{{\pi d_{c}^{2} }}$$

Here, *F*_*b*_ and *d*_*c*_ are the axial loading force and cylinder diameter of the confined compaction device, respectively.

**Fig. 4:**
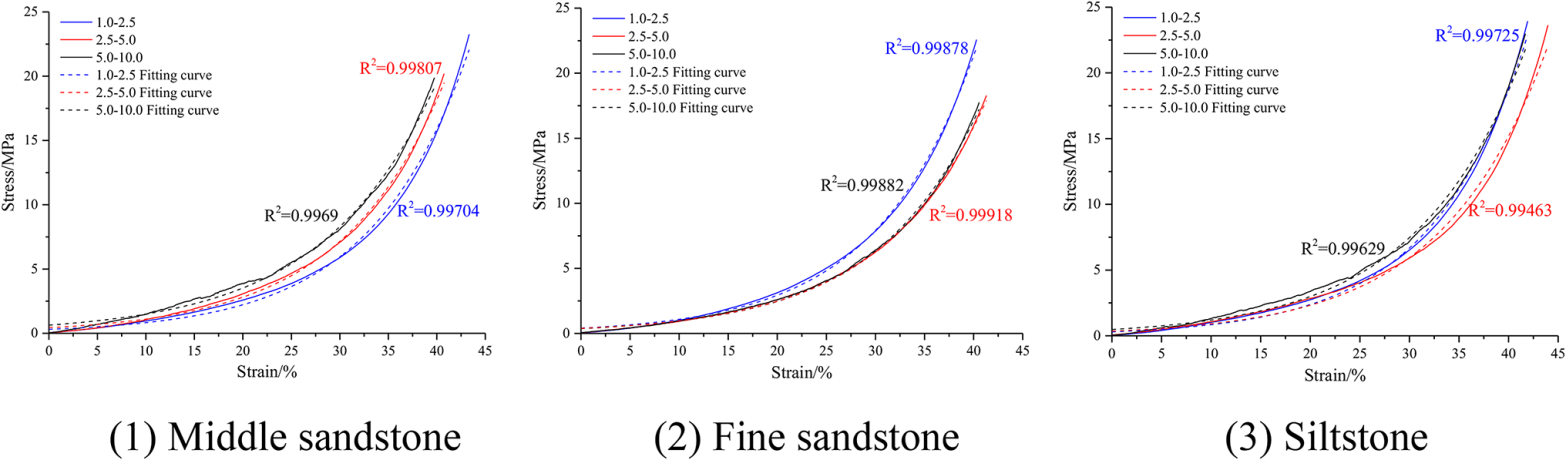
Stress–strain curves of mono-sized fragmented rock.

Fragmented rock mass particles (250 g) of medium sandstone, fine sandstone, and siltstone with different size ranges were placed in a lateral pressure-limiting compaction device (to form a standard sample), as shown in Fig. 3. Compression tests conducted via an MTS electro-hydraulic servo system under displacement control (0.8 mm/min) yielded the compaction strength values (Table 3) and corresponding stress–strain profiles (Fig. 4) for mono-sized particulate samples. Strength values were standardized at 65 mm axial displacement for 250 g specimens to ensure comparability.

**Table 3:** Compaction strength of mono-sized fragmented rock.

Lithology	1.0–2.5 mm	2.5–5.0 mm	5.0–10.0 mm
Middle sandstone	23.26 MPa	20.19 MPa	19.87 MPa
Fine sandstone	22.57 MPa	18.27 MPa	17.74 MPa
Siltstone	23.92 MPa	23.63 MPa	22.98 MPa

Analysis of particulate rock aggregates under confined compression (Table 3 and Fig. 4) revealed stress-dependent axial strain progression with progressively diminishing increments. Larger particle size fractions exhibited reduced compressive resistance across identical lithologies. Given the irrecoverable structural deformation inherent to granular media, cyclic load-unload analysis was deemed negligible compared to constrained compaction studies. Stress–strain relationships for uniform-sized specimens followed exponential patterns, mathematically described by Eq. (4), with statistical validation parameters detailed in Table 4.

4 $$\sigma_{y} = a_{1} e^{{b_{1} \varepsilon_{y} }}$$

**Table 4:** Fitting parameters of the compaction stress–strain curve for mono-sized fragmented rock.

Lithology	Particle size range (mm)	Fit parameters *a*_1_	Fit parameters *b*_1_	Fitting R^2^
Middle sandstone	1.0–2.5	0.31123	0.09832	0.99704
	2.5–5.0	0.43567	0.09327	0.99807
	5.0–10.0	0.63606	0.08563	0.9969
Fine sandstone	1.0–2.5	0.40384	0.09992	0.99878
	2.5–5.0	0.38467	0.09299	0.99918
	5.0–10.0	0.37213	0.09457	0.99882
Siltstone	1.0–2.5	0.30168	0.10322	0.99725
	2.5–5.0	0.35506	0.09154	0.99463
	5.0–10.0	0.47938	0.09154	0.99629

Here, *σ*_*y*_ and *ε*_*y*_ denote axial stress and strain during confined compression of particulate rock aggregates, with a1 and b1 serving as model calibration coefficients.

The compaction coefficient of particulate rock aggregates is defined as the volumetric reduction during confined compression relative to initial volume, expressed mathematically as:

5 $$K_{ys} = \frac{{\Delta V_{y} }}{{V_{0}^{\prime } }}$$

Under constant cross-sectional area conditions during confined compression, Eq. (5) simplifies to:

6 $$K_{ys} = \frac{{\Delta V_{y} }}{{V_{0}^{\prime } }}{ = }\frac{{\Delta h_{y} }}{{h_{y} }} = \varepsilon_{y}$$

Here, *K*_*ys*_ is the compaction coefficient of the fragmented rock, ∆*V*_*y*_ is the volume reduction during compaction, *h*_*y*_ and ∆*h*_*y*_ are the heights of the fragmented rock before side-limited compaction loading and the height reduction during compaction, respectively.

This equivalence reveals that particulate densification corresponds directly to axial strain. Stress-dependent progression exhibited incremental strain accumulation with progressively attenuated growth rates.

### Pre-/post-compaction mass variations

Post-compaction analysis of uniform-sized particulate aggregates revealed granulometric redistribution (Table 5), with total recovered mass marginally reduced due to handling losses. Despite universal particle size reduction, the original size fraction (5.0–10.0 mm) remained dominant at approximately 40% mass proportion, though cumulative smaller fractions exceeded this value. Compaction-induced stress concentration at interparticle contacts preferentially fragmented angular protrusions, generating fines that occupied void spaces—a key densification mechanism through pore infilling and particle rearrangement.

**Table 5:** Different particle size quality of mono-sized fragmented rock after compaction.

Before compaction	1.0–2.5 mm				2.5–5.0 mm				5.0–10.0 mm			
After compaction	0–1	1–2.5	2.5–5	5–10	0–1	1–2.5	2.5–5	5–10	2.5–5	1–2.5	2.5–5	5–10
Middle sandstone	131.2	118.4	–	–	74.9	67.8	106.9	–	45.5	32	69.7	102.8
Proportion/%	52.48	47.36	–	–	29.96	27.12	42.76	–	18.20	12.80	27.88	41.12
Fine sandstone	144.5	105.4	–	–	78.9	80.4	90.7	–	43.2	40.1	69.1	97.6
Proportion/%	57.80	42.16	–	–	31.56	32.16	36.28	–	17.28	16.04	27.64	39.04
Siltstone	152.3	97.4	–	–	89.8	52.9	106.9	–	59.3	28	55.3	107.4
Proportion/%	60.92	38.96	–	–	35.92	21.16	42.76	–	23.72	11.20	22.12	42.96

### Characteristics of porosity change

Granular rock aggregates constitute polydisperse particulate systems classified as porous media, where intergranular voids dominate over matrix microporosity. The effective porosity—defined as interparticle void volume fraction—is governed by particle morphology, gradation, and packing configuration. This study specifically examines porosity evolution under confined compression conditions.

The volume before and after rock fragmentation can be expressed as:

7 $$V_{0} = \frac{m}{\rho }$$

8 $$V_{0}^{\prime } = A_{c} h_{y} = \frac{{\pi d_{c}^{2} }}{4}h_{y}$$

The initial porosity of the fragmented rock can be expressed as follows:

9 $$\varphi_{0} = \frac{{V_{0}^{\prime } - V_{0} }}{{V_{0}^{\prime } }}{ = }1 - \frac{m}{{\rho A_{c} h_{y} }}$$

In the process of confined compaction, the height and porosity of the fragmented rock under different stress states can be expressed as follows:

10 $$h = h_{y} - \Delta h_{y}$$

11 $$\varphi = 1 - \frac{m}{{\rho A_{c} (h_{y} - \Delta h_{y} )}}{ = }1 - \frac{4m}{{\rho \pi d^{2} (h_{y} - \Delta h_{y} )}}$$

Here, *m* and *ρ* are the mass and density of the rock sample, *A*_*c*_ is the cross-sectional area of the cylinder, *φ*_0_ is the initial porosity of the confined compaction of the fragmented rock.

**Fig. 5:**
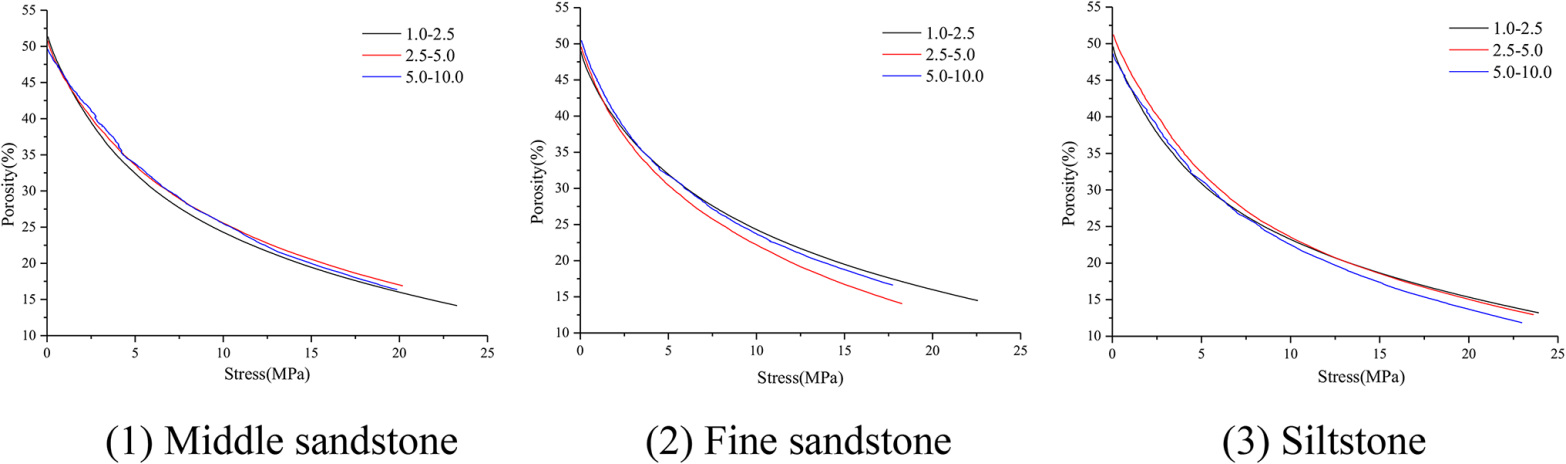
Porosity curve of mono-sized fragmented rock.

The confined compaction porosity of the fragmented rock with mono-sized under different stress states was calculated using Eq. (11). Figure 5 shows porosity change curve. With an increase in the stress, the porosity and its reduction range gradually decrease. The initial porosity, porosity after compaction, and porosity reduction range are approximately 50%, 15%, and 35%, respectively. Under the action of axial pressure, the fragmented rock overcomes the frictional resistance between particles and slides or rolls to a relative equilibrium position that is more dense and stable, and the equilibrium position will gradually change with the increase in the axial pressure, that is, the equilibrium is a dynamic equilibrium, and the fragmented rock mass will no longer shift until the pores between the fragmented rock are small enough, thus gradually reducing the pore volume of the fragmented rock. The porosity decreases gradually.

### Acoustic emission characteristics

**Fig. 6:**
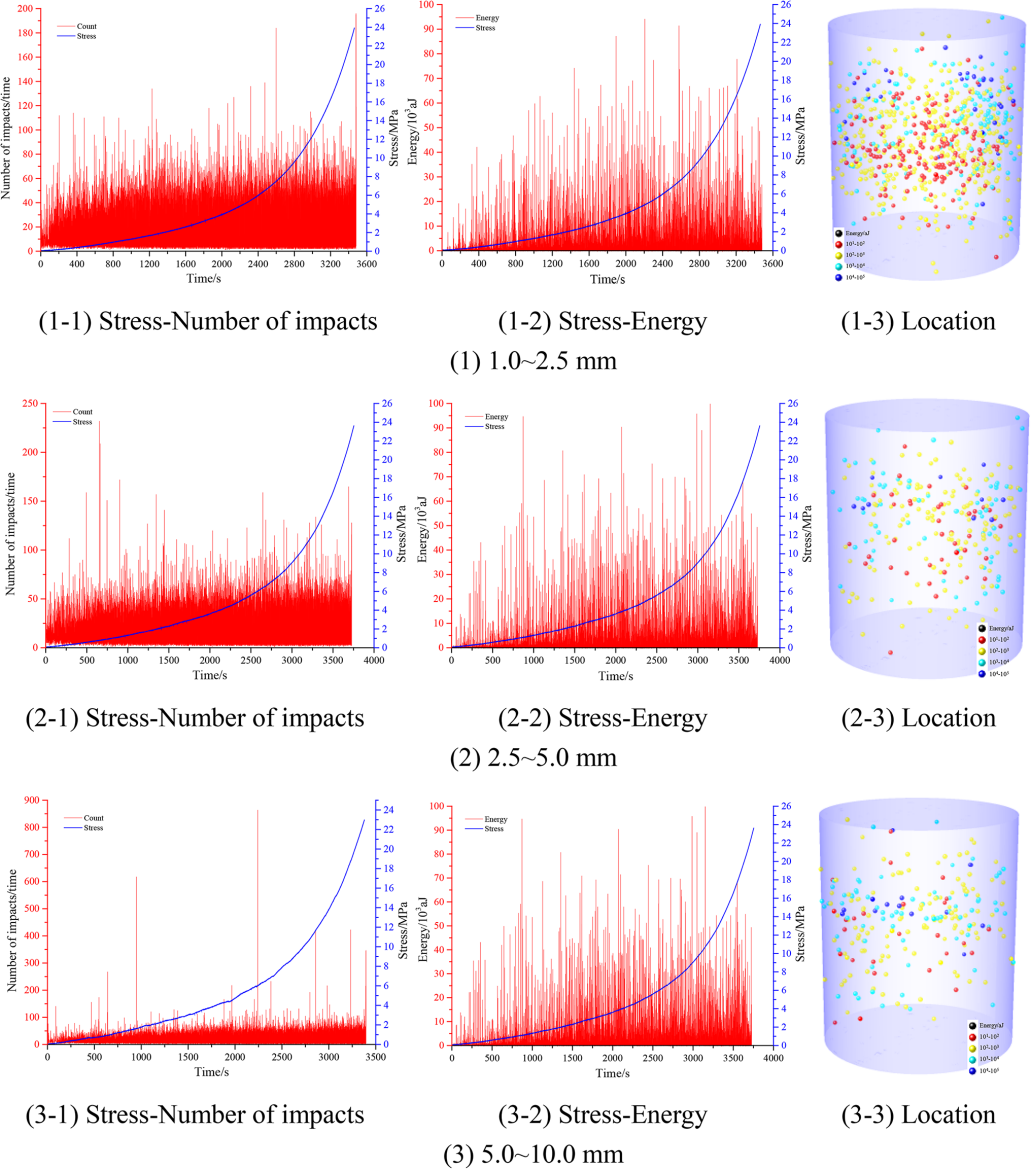
AE characteristics of confined compaction of mono-sized fragmented siltstone.

Real-time acoustic emission (AE) monitoring during particulate compaction employed an 8-channel Express-8 system (Physical Acoustics Corp.) with 50 dB threshold. Probes affixed externally (Fig. 3 positioning) underwent pre-test validation via pencil-lead fracture checks. Analysis of uniform siltstone aggregates revealed continuous AE activity throughout compression, contrasting conventional rock testing where signals concentrate near peak strength. Stress-dependent progression showed escalating event frequency (the average number of impacts is approximately 15 hits/s) and energy release (the average energy is approximately 0.3 × 10^3^ aJ/s), indicating intensifying interparticle interactions. Spatial AE distribution demonstrated preferential central and upper zone localization (Fig. 6). This vertical compaction gradient-evidenced by sparse lower-region signals-confirms top-down densification mechanics. AE signatures effectively traced particulate reorganization and fragmentation dynamics, validating their utility for real-time compaction monitoring.

### Confined compaction stage of fragmented rock

The ratio of the axial stress to the axial strain in the compaction process of fragmented rock is called the compression modulus. The compression modulus for the fragmented rock can be expressed as follows:

12 $$E_{y} = \frac{{\sigma_{y} }}{{\varepsilon_{y} }}$$

Here, *E*_*y*_ is the compression modulus of the fragmented rock.

**Fig. 7:**
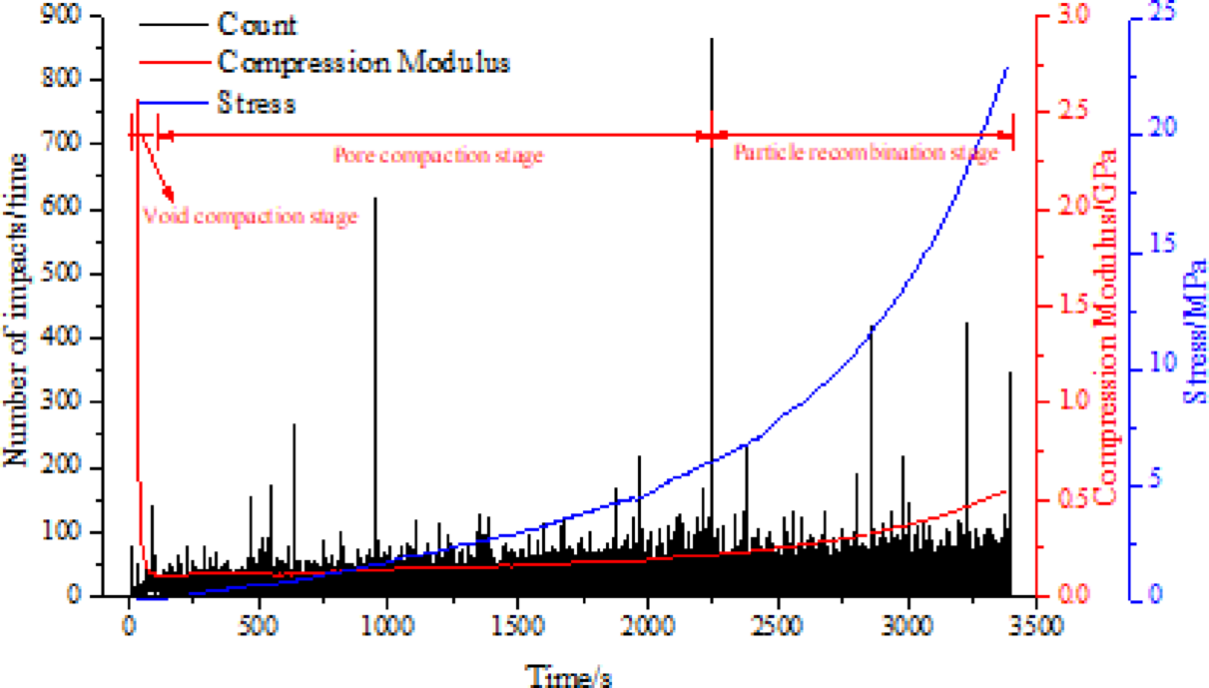
Compaction stage division of fragmented rock.

The confined compaction of 5.0–10.0 mm siltstone aggregates was analyzed through stress progression, acoustic emission (AE) events, and compression modulus evolution (Fig. 7). Three distinct densification phases were identified: void compaction stage, pore compaction stage, and particle recombination stage. Void compaction stage: Rapid modulus decline during brief stress application, dominated by macro-void closure without particle interaction. Pore compaction stage: Linear modulus recovery as meso-pores compaction under sustained stress, triggering particle fragmentation and fines migration. Particle recombination stage: Nonlinear modulus escalation from micro-scale particulate rearrangement, forming metastable configurations under confinement conditions. Spatiotemporal AE patterns confirmed progressive top-down compaction, with energy release correlating to pore hierarchy reduction. Void-pore differentiation was particle-size-referenced: voids exceeded initial grain dimensions, while pores formed interstitial spaces.

## Compaction characteristics of fragmented coal with mono-sized

### Compaction strength

**Fig. 8:**
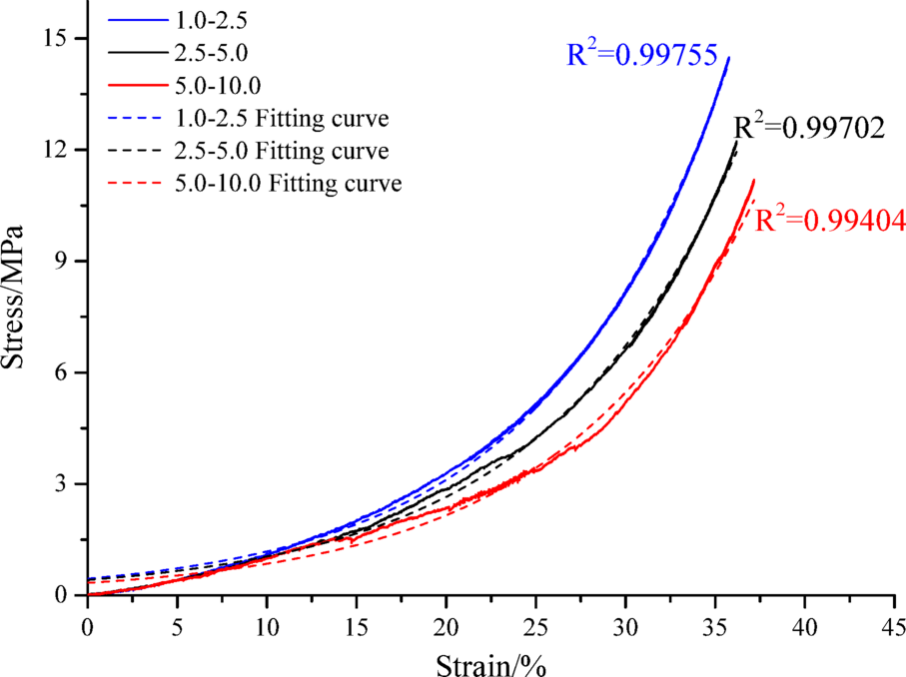
Stress–strain curve of the confined compaction of mono-sized fragmented coal.

Lateral compaction tests on 150 g particulate coal specimens established size-dependent strength parameters at 45 mm axial displacement (Table 6 and Fig. 8). Results demonstrated stress-progressive strain accumulation with incrementally diminishing rates. Compressive resistance exhibited inverse correlation with particle size, while equivalent stress conditions produced greater strain in larger particle size.

**Table 6:** Compaction strength of mono-sized fragmented coal.

Lithology	1.0–2.5 (mm)	2.5–5.0 (mm)	5.0–10.0 (mm)
Fragmented coal	14.49 MPa	12.23 MPa	11.18 MPa

### Pre-/post-compaction mass variations

Post-compaction granulometric analysis of uniform-sized coal particulates (Table 7) revealed hierarchical fragmentation patterns: directional compression reduced all particles below initial dimensions, yet the original size class retained dominance (≈40% mass retention). Cumulative smaller fractions exceeded the original size proportion, confirming preferential particle comminution during pore-space infilling.

**Table 7:** Quality of mono-sized fragmented coal after compaction.

Before compaction	1–2.5				2.5–5				5–10			
After compaction	0–1	1–2.5	2.5–5	5–10	0–1	1–2.5	2.5–5	5–10	0–1	1–2.5	2.5–5	5–10
Coal	76.2	73.5	–	–	46.5	42.7	60.4	–	32.7	24.5	30.6	62.1
Proportion/%	50.80	49.00	–	–	31.00	28.47	40.27	–	21.80	16.33	20.40	41.40

### Characteristics of porosity change

**Fig. 9:**
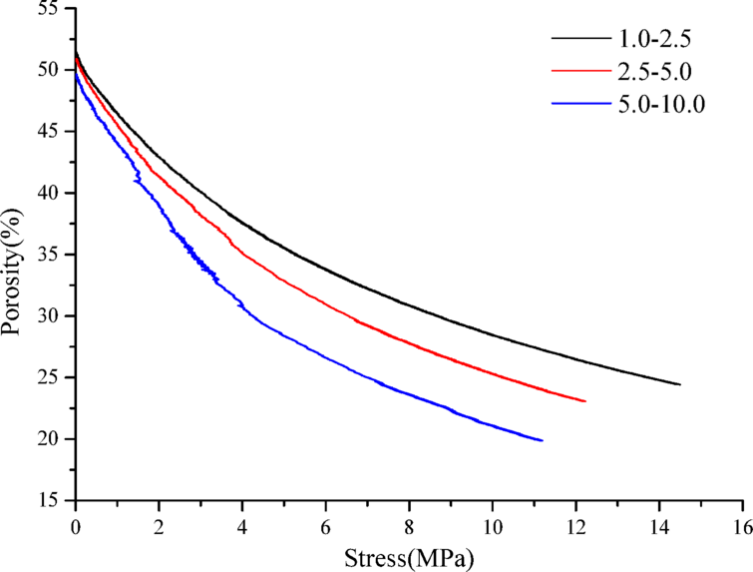
Porosity curve of mono-sized fragmented coal under confined compaction.

The confined compaction porosity of mono-sized fragmented coal in different stress states was calculated using Eq. (11). Figure 9 shows its variation curve. As shown, with increasing stress, the porosity and its reduction range gradually decrease. The initial porosity, porosity after compaction, and porosity reduction range were approximately 50%, 20%, and 30%, respectively. Under the same stress condition, with an increase in the particle size range of the fragmented coal, the lower the porosity, the greater the reduction range.

### Acoustic emission characteristics

**Fig. 10:**
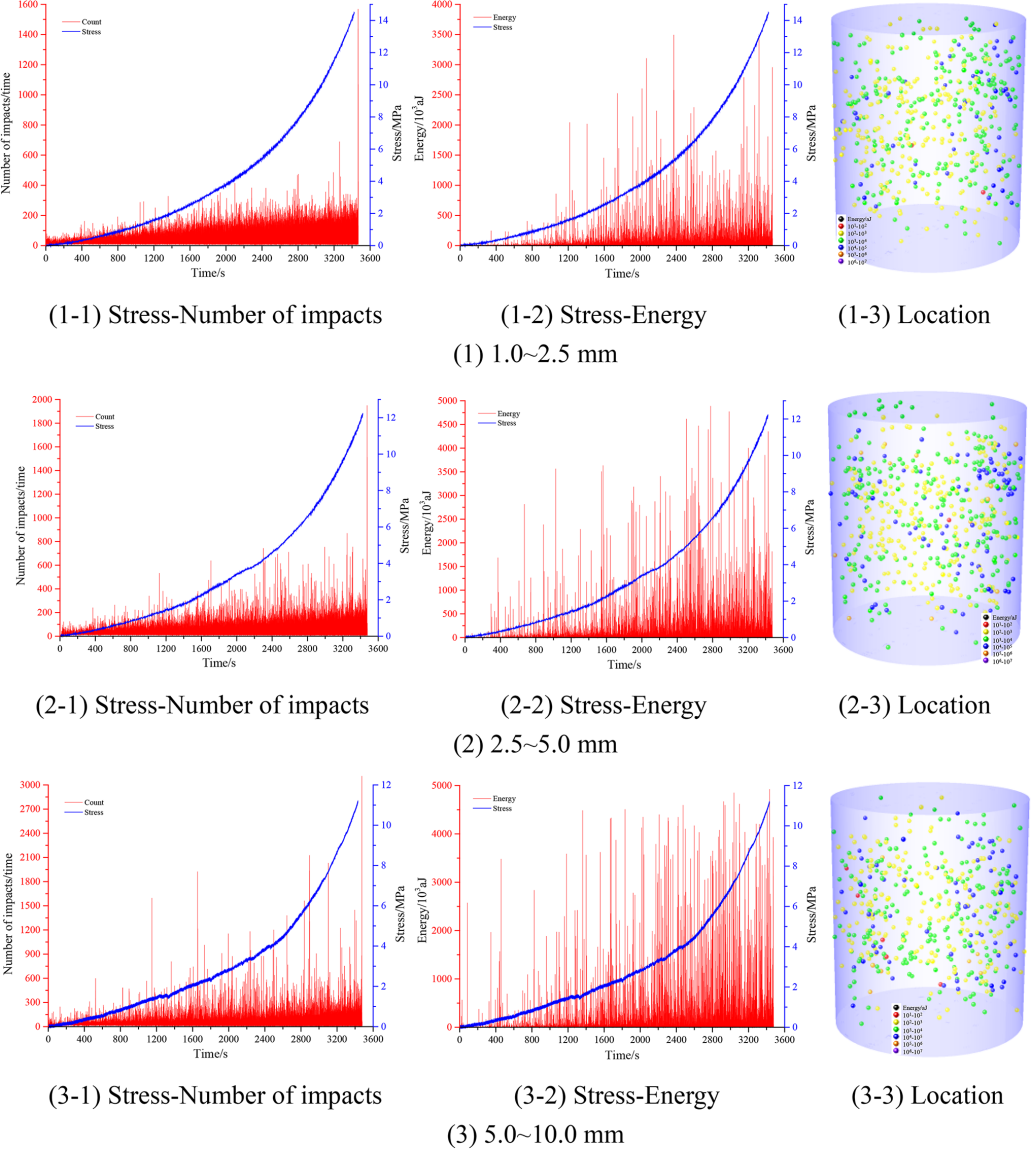
AE characteristics of the confined compaction of mono-sized fragmented coal.

Figure 10 shows the stress–impact number, stress–energy, and AE localization in the confined compaction process of the mono-sized fragmented coal. As shown, with the loading of the stress, the number of AE impacts generated by the extrusion between fragmented coal bodies gradually increased (the average number of impacts was 40 times/s), and the energy gradually increased (the average energy range was 5.0 × 10^3^ aJ/s), that is, the gradual loading of the stress intensified the extrusion between fragmented coal bodies. Acoustic emission (AE) monitoring revealed higher impact frequencies and greater energy release in uniform coal particulates compared to rock aggregates under equivalent compression, attributed to coal’s reduced compressive strength facilitating intensified interparticle stress transfer. Spatial AE location demonstrated particle size-dependent energy gradients: coal specimens exhibited centralized AE localization (most events within core regions). With an increase in the particle size range of the fragmented coal, the number of low-energy position points gradually decreased. This core-dominated energy dissipation pattern, contrasted with rock’s dispersed AE distribution, confirms enhanced mechanical interactions near specimen centroids during coal densification.

## Conclusions

A laboratory investigation examined the compressive properties of mono-sized fragmented coal and rock aggregates using a custom-designed lateral pressure limiting compaction device. Multi-parameter analysis evaluated compressive strength, pre-/post-compaction mass variations, porosity evolution, and acoustic emission (AE) signatures across distinct particle size.

1. Bulking coefficient testing revealed an inverse correlation between particle dimensions and bulking coefficients. Compressive strength similarly decreased with increasing particle size ranges, attributed to reduced interparticle contact density and stress distribution inefficiencies.
2. Axial strain demonstrated logarithmic growth under progressive loading, accompanied by diminishing porosity reduction rates. Concurrent AE monitoring captured proportional increases in event frequency and energy release, reflecting intensified particle interactions.
3. Post-compaction particle redistribution: Post-densification granulometry showed retained dominance of original size fractions (approximately 40%) despite universal comminution. Cumulative smaller particles exceeded original fractions, confirming hierarchical fragmentation mechanisms during pore infilling.
4. Coal particulates generated higher AE activity than rock counterparts due to reduced compressive strength. Three- stage densification emerged: void compaction stage (rapid void closure), pore compaction stage (progressive interstitial reduction), and particle recombination stage (microstructural rearrangement with energy dissipation).

## Data Availability

Data are available from the corresponding author on reasonable request.

## Declarations

### Permission statement

We have obtained permission to collect samples from Nanliang Coal Mine. The coal samples used in our experiment were all taken from the 3–1 coal seam of the mine, and the rock samples were taken from the roof and bottom of the 3–1 coal seam of the mine.

## References

[1] Wang, Z.-Q. et al. Method of division and engineering use of “three band” in the stope again. *J. China Coal Soc.* **38**, 287–293 (2013).

[2] Jiang, L.-S., Wu, Q.-S., Li, X. & Ding, N. Numerical simulation on coupling method between mining-induced stress and goaf compression. *J. China Coal Soc.* **42**, 1951–1959 (2017).

[3] Zhu, D.-F., Tu, S.-H., Yuan, Y., Ma, H.-S. & Li, X.-Y. An approach to determine the compaction characteristics of fractured rock by 3D discrete element method. *Rock Soil Mech.* **39**, 1047–1055 (2018).

[4] Huang, Y. et al. Numerical simulation study on macroscopic mechanical behaviors and micro-motion characteristics of gangues under triaxial compression. *Powder Technol.* **320**, 668–684 (2017).

[5] Fan, L. & Liu, S. A conceptual model to characterize and model compaction behavior and permeability evolution of broken rock mass in coal mine gobs. *Int. J. Coal Geol.* **172**, 60–70 (2017).

[6] Zhang, C., Zhao, Y.-X., Tu, S.-H. & Zhang, T. Numerical simulation of compaction and re-breakage characteristics of coal and rock samples in goaf. *Chin. J. Geotech. Eng.* **45**, 696–704 (2020).

[7] Zhang, C., Ren, Z., Hao, D. & Zhang, T. Numerical simulation of particle size influence on the breakage mechanism of broken coal. *Arab. J. Sci. Eng.* **45**, 9171–9185 (2020).

[8] Zhang, C., Liu, J., Zhao, Y., Han, P. & Zhang, L. Numerical simulation of broken coal strength influence on compaction characteristics in goaf. *Nat. Resour. Res.* **29**, 2495–2511 (2020).

[9] Huang, Z. et al. A numerical study of macro-mesoscopic mechanical properties of gangue backfill under biaxial compression. *Int. J. Min. Sci. Technol.* **26**, 309–317 (2016).

[10] Liu, Z., Zhou, N. & Zhang, J. Random gravel model and particle flow based numerical biaxial test of solid backfill material. *Int. J. Min. Sci. Technol.* **23**, 463–467 (2013).

[11] Ma, Z.-G., Guo, G.-L., Chen, R.-H. & Mao, X.-B. An experimental study on the compaction of water-saturated over-broken rock. *Chin. J. Rock Mech. Eng.* **24**, 1139–1144 (2005).

[12] Pan, D. et al. Influence characteristics and mechanism of fragmental size of broken coal mass on the injection regularity of silica sol grouting. *Constr. Build. Mater.* **269**, 121251 (2021).

[13] Qin, Y., Xu, N., Guo, Y., Li, J. & Han, W. Physical simulation of the influence of the original rock strength on the compaction characteristics of caving rock in longwall goaf. *R. Soc. Open Sci.* **9**, 220558 (2022). 36117867 10.1098/rsos.220558 PMC9470253

[14] Wen, P. et al. Compaction deformation and acoustic emission characteristics of fractured rock with different lithology in goaf. *J. Min. Saf. Eng.* **41**, 384–394 (2024).

[15] Xu, J.-Y. & Liu, S. Research on fractal characteristics of marble fragments subjected to impact loading. *Rock Soil Mech.* **33**, 3225–3229 (2012).

[16] Wang, L., Yin, M., Kong, H. & Zhang, H. Experimental study on breakage characteristics and energy dissipation of the crushed rock grains. *KSCE J. Civ. Eng.* **26**, 1465–1478 (2022).

[17] Zhang, J., Wang, H., Chen, S. & Li, Y. Bearing deformation characteristics of large size broken rock. *J. China Coal Soc.* **43**, 1000–1007 (2018).

[18] Li, B., Liang, Y., Zhang, L. & Zou, Q. Breakage law and fractal characteristics of broken coal and rock masses with different mixing ratios during compaction. *Energy Sci. Eng.* **7**, 1000–1015 (2019).

[19] Li, B. et al. Experimental research on the influence of different factors on the behaviour of broken coal and rock particles during compaction. *Constr. Build. Mater.* **367**, 130127 (2023).

[20] Chu, T.-X., Li, P., Chao, J.-K., Yu, M.-G. & Han, X.-F. Bulking coefficient evolution characteristics and mechanism of compacted broken coal. *J. China Coal Soc.* **42**, 3182–3188 (2017).

[21] Hao, D. Y., Tu, S. H., Zhang, L., Zhao, H. B. & Xu, S. K. Experimental study on characteristics of gas seepage in broken coal and rock. *Energy Sci. Eng.* **12**, 4737–4752 (2024).

[22] Su, C.-D., Gu, M., Tang, X. & Guo, W.-B. Experiment study of compaction characteristics of crushed stones from coal seam roof. *Chin. J. Rock Mech. Eng.* **31**, 18–26 (2012).

[23] Yavuz, H. An estimation method for cover pressure re-establishment distance and pressure distribution in the goaf of longwall coal mines. *Int. J. Rock Mech. Min. Sci.* **41**, 193–205 (2004).

[24] Zhang, Z.-N., Miao, X.-X. & Ge, X.-R. Testing study on compaction breakage of loose rock block. *Chin. J. Rock Mech. Eng.* **24**, 451–455 (2005).

[25] Zhu, D. et al. Modeling and calculating for the compaction characteristics of waste rock masses. *Int. J. Numer. Anal. Methods Geomech.* **43**, 257–271 (2019).

[26] Zhang, C. et al. Influence mechanism of particle size on the compaction and breakage characteristics of broken coal mass in goaf. *J. China Coal Soc.* **45**, 660–670 (2020).

[27] Zhang, C., Zhao, Y. & Bai, Q. 3D DEM method for compaction and breakage characteristics simulation of broken rock mass in goaf. *Acta Geotech.* **17**, 2765–2781 (2022).

[28] Zhang, C., Li, B., Song, Z., Liu, J. & Zhou, J. Breakage mechanism and pore evolution characteristics of gangue materials under compression. *Acta Geotech.* **17**(11), 4823–4835 (2022).

[29] Wang, Z.-H., Liu, P.-J., Sun, W.-C., Shui, Y.-T. & Zhong, Q. Study on the compressive deformation and load-bearing capacity of broken blocks of coal and rock. *J. Min. Saf. Eng.* **40**, 599–610 (2023).

[30] Miao, X.-X., Mao, X.-B., Hu, G.-W. & Ma, Z.-G. Research on broken expand and press solid characteristics of rocks and coals. *J. Exp. Mech.* **12**, 394–400 (1997).

[31] Yu, B.-Y., Chen, Z.-Q., Feng, M.-M., Wu, J.-Y. & Ding, Q.-L. Microstructure evolution of saturated crushed limestone under lateral confined compression based on CT test. *J. China Coal Soc.* **42**, 367–372 (2017).

